# Cumulative exposure to integrase inhibitors and metabolic factors as independent predictors of severe hepatic steatosis in people with HIV

**DOI:** 10.1093/jac/dkag351

**Published:** 2026-10-21

**Authors:** Francesca Panza, Manuel Crispo, Margherita Sambo, Alessandra Bailoni, Enrico Morelli, Gabriele Giuliano, Daniela Romano, Francesco Pippi, Michele Trezzi, Francesca Montagnani, Mario Tumbarello, Massimiliano Fabbiani

**Affiliations:** Department of Medical Biotechnologies, University of Siena, Siena, Italy; Infectious and Tropical Diseases Unit, Siena University Hospital, Siena, Italy; Department of Medical Biotechnologies, University of Siena, Siena, Italy; Infectious and Tropical Diseases Unit, Siena University Hospital, Siena, Italy; Department of Medical Biotechnologies, University of Siena, Siena, Italy; Infectious and Tropical Diseases Unit, Siena University Hospital, Siena, Italy; Department of Medical Biotechnologies, University of Siena, Siena, Italy; Infectious and Tropical Diseases Unit, Siena University Hospital, Siena, Italy; Department of Medical Biotechnologies, University of Siena, Siena, Italy; Infectious and Tropical Diseases Unit, Siena University Hospital, Siena, Italy; Department of Medical Biotechnologies, University of Siena, Siena, Italy; Infectious and Tropical Diseases Unit, Siena University Hospital, Siena, Italy; Department of Medical Biotechnologies, University of Siena, Siena, Italy; Infectious and Tropical Diseases Unit, Siena University Hospital, Siena, Italy; Infectious and Tropical Diseases Unit, Siena University Hospital, Siena, Italy; Department of Medical Biotechnologies, University of Siena, Siena, Italy; Infectious and Tropical Diseases Unit, Siena University Hospital, Siena, Italy; Department of Medical Biotechnologies, University of Siena, Siena, Italy; Infectious and Tropical Diseases Unit, Siena University Hospital, Siena, Italy; Department of Medical Biotechnologies, University of Siena, Siena, Italy; Infectious and Tropical Diseases Unit, Siena University Hospital, Siena, Italy

## Abstract

**Background:**

The progressive ageing of people living with HIV (PLWH) has increased hepatic and metabolic comorbidities.

**Objectives:**

This study evaluates the impact of HIV-related, metabolic and behavioural factors on hepatic steatosis and fibrosis development using vibration-controlled transient elastography (VCTE).

**Methods:**

This is a single-centre cross-sectional study evaluating an Italian cohort of PLWH at an academic medical centre. Steatosis (via CAP, dB/m) and fibrosis (kPa) were assessed using VCTE. Lifestyle habits were investigated through a structured questionnaire. Factors associated with severe steatosis (S3) and with fibrosis were analysed.

**Results:**

Overall, 265 patients were included (median age 53.9 years; 89.4% with HIV-RNA < 50 copies/mL; median CD4 count 758 cells/mm^3^). Moderate-to-severe steatosis (S2–S3) affected 49% of the cohort (S3: 28.5%). In multivariate analysis, independent predictors of severe (S3) steatosis were longer cumulative integrase inhibitor (InSTI) exposure (aOR 1.19/year, *P* = 0.002), higher BMI (aOR 1.45 per kg/m^2^, *P* < 0.001), daily alcohol consumption (aOR 2.41, *P* = 0.031) and higher LDL cholesterol (aOR 1.20 per +10 mg/dL, *P* = 0.003). Regular physical activity was associated with lower CAP. Higher CAP values showed an independent association with increased liver stiffness (mean change +0.07 kPa per +10 db/m, *P* = 0.016), while virological suppression (HIV-RNA < 50 copies/mL) was significantly associated with reduced liver stiffness (mean change −1.61 kPa, *P* = 0.001).

**Conclusions:**

Hepatic steatosis and fibrosis are highly prevalent in PLWH. Cumulative InSTI exposure and metabolic factors may drive severe steatosis, which may accelerate fibrotic damage, whereas effective virological suppression can significantly lower fibrosis risk. Strict metabolic monitoring and lifestyle interventions should be integrated into PLWH long-term care protocols.

## Introduction

Since the advent of antiretroviral therapy (ART), HIV infection has evolved into a chronic condition, enabling people living with HIV (PLWH) to achieve prolonged survival and progressive ageing. As a result, age-related comorbidities, increasingly recognized in the general population, are also observed in PLWH, who represent a category at increased risk of metabolic, liver and cardiovascular diseases due to both HIV- and non-HIV-related factors. Indeed, the prevailing focus in contemporary debates within the HIV medical community has progressively shifted towards metabolic complications, including weight gain and obesity, which have become increasingly prevalent in this population.^1^

Excess weight gain, especially central adiposity, is a key driver of ectopic fat accumulation, including hepatic fat deposition. This process contributes to the development of metabolic dysfunction-associated steatotic liver disease (MASLD), nowadays the most common chronic liver disease worldwide with an expected increasing prevalence.^2^ Hepatic steatosis represents the initial step in a progressive spectrum of liver diseases, ranging from simple fat accumulation (metabolic dysfunction-associated steatotic liver, MASL) to metabolic dysfunction-associated steatohepatitis (MASH), progressive liver fibrosis, cirrhosis and ultimately to MASH-related hepatocellular carcinoma (HCC). In this context, hepatic steatosis can be considered the primum movens of liver disease progression, underscoring the importance of its early detection and characterization.

Notably, PLWH are exposed to multiple and potentially interacting risk factors for hepatic steatosis, including HIV-related factors (such as chronic immune activation and long-term ART exposure), metabolic conditions (including obesity, insulin resistance and dyslipidaemia), as well as lifestyle and behavioural factors such as diet and alcohol consumption. In the context of ART, integrase strand transfer inhibitors (InSTI) and tenofovir alafenamide have been associated to significant weight gain, raising concerns about their potential long-term metabolic consequences.^3–7^ However, the relative contribution and interplay of these determinants remain poorly understood.

As the burden of MASLD in PLWH is growing—with prevalence estimates ranging between 20% and 38% in Western cohorts receiving stable ART and reaching up to 40% among individuals over 50 years of age or with long-term ART exposure^8^—liver disease remains a major cause of morbidity and mortality in this population, accounting for approximately 9% of deaths between 2010 and 2023. Indeed, while advances in screening and treatment have significantly reduced HBV- and HCV-related mortality, the rise of metabolic-related liver disease ensures it remains a leading cause of death in this population.^9^

Hepatic vibration-controlled transient elastography (VCTE) has been extensively validated as a reliable, non-invasive method for assessing liver steatosis and fibrosis in HIV-uninfected population.^10^ Its application in PLWH has been reported,^11^ and recent European AIDS Clinical Society (EACS) guidelines recommend its use in PLWH in this setting.^12^ However, data integrating this parameter with comprehensive assessments of metabolic profiles and lifestyle factors remain limited.^13^

## Materials and methods

### Objectives

The aim of this study was to investigate the relative contribution of HIV-related, metabolic and behavioural factors to the development of hepatic steatosis in PLWH, using VCTE as a non-invasive assessment tool.

### Study design

We conducted a single-centre cross-sectional study, enrolling PLWH who were on chronic follow-up at the outpatient HIV Clinic of the University Hospital of Siena and underwent transient elastography and metabolic assessment under fasting condition in routine clinical practice during years 2024 and 2025.

Data collection was approved by the local ethics committee, and informed consent was obtained from all patients before participation. The study was carried out in accordance with the ethical principles of the Declaration of Helsinki and with the Good Clinical Practice guidelines of the International Conference on Harmonization.

Enrolled patients were selected based on the following inclusion criteria: age > 18 years, documented HIV-1 infection and on regular follow-up at our clinic (at least one visit in the previous year). Exclusion criteria were active viral hepatitis from HAV (defined as positive IgM for HAV), HBV (defined as positive plasma HBV-DNA), HCV (defined as positive plasma HCV-RNA), excessive alcohol consumption (defined as alcohol intake > 50 g/day for women and >60 g/day for men),^2^ current ascitic decompensation, pregnancy and unsuitable morphotype for hepatic elastography with M probe.

### Clinical, anthropometric and laboratory data collection

Enrolled patients underwent a metabolic-clinical evaluation during a routine check-up at our HIV clinic. Assessment included comprehensive blood chemistry tests, liver VCTE performed with FibroScan^®^ with detection of hepatic fibrosis (kPa) and steatosis (dB/m), blood pressure measurement with manual sphygmomanometer (mmHg), measurement of body weight (kg), height (cm), BMI (kg/m^2^) and waist and hip circumferences (cm). Abnormal waist circumference was defined as >102 cm in men and >88 cm in women. Similarly, a pathological waist-to-hip ratio was defined as >0.90 in men and >0.85 in women.^14^ Moreover, collection of patients’ lifestyle habits was performed through a standardized questionnaire, as specified below. Clinical and viro-immunological variables and concomitant therapies were obtained by patients’ interview and reviewing patients’ records. To calculate the cumulative exposure to each drug class, we summed the total duration (in years) during which patients were exposed to at least one molecule belonging to that class, excluding any treatment interruptions. All molecules within the same class were analysed collectively, with the exception of thymidine analogues and tenofovir alafenamide, which were also evaluated separately.

### Hepatic assessment

Transient elastography was performed by trained physicians using VCTE (FibroScan^®^ 502 touch, by Echosens, Paris, France), estimating liver fibrosis by measuring liver stiffness (expressed in kPa) and hepatic steatosis by measuring controlled attenuation parameter (CAP) (expressed in dB/m). At least 10 measurements were obtained for each patient using the M-probe. Liver stiffness and CAP values were expressed as the median of all valid measurements (at least 10 measurements were collected for each patient). To minimize operator-associated bias, the measurement was conducted in strict adherence to both the protocol and the manufacturer’s instructions.

Hepatic steatosis was classified into three categories according to the following threshold values: grade zero steatosis (S0) < 238 dB/m, grade 1 steatosis (S1) 238–259 db/m, grade 2 steatosis (S2) 260–291 dB/m and grade 3 steatosis (S3) >292 dB/m.^13^

Hepatic fibrosis was considered significant if liver stiffness was >8 kPa.^12^

The MASLD has been defined according to the criteria of the most recent EASL-EASDL-EASO guidelines.^2^

The homeostatic model for the assessment of insulin resistance (HOMA-IR) was calculated in fasting patient and was defined as a HOMA-IR value > 2.5.^15,16^

### Lifestyle habits evaluation

An anonymous structured questionnaire was used to assess patients’ self-reported lifestyle habits. The variables investigated were selected from those that, according to the literature, were mostly associated with liver steatosis and fibrosis. In particular, the following variables were collected: (i) smoking habits: non-smokers/active smokers and average number of cigarettes/days; (ii) alcohol consumption habit: no alcohol consumption, daily consumption, once/week or less consumption; (iii) physical activity frequency: no routinary physical activity, one time per week, more than one time/week; and (iv) sedentary versus non-sedentary work.

All data collected were anonymized by a unique alphanumeric code for each patient and grouped in a single database.

### Cardiovascular risk assessment

Participants’ cardiovascular risk was estimated by measuring resting blood pressure with a manual sphygmomanometer and assessing the 10-year cardiovascular risk with the American Heart Association (AHA) 10-year Atherosclerotic Cardiovascular Disease (ASCVD) score.^17^ Family history of major cardiovascular events (defined as myocardial infarction, stroke or other major cardiovascular events, occurring before age 55 in male first-degree relatives and before 65 in female first-degree relatives^18^) was also explored.

### Statistical analysis

Descriptive statistics (number, proportion, median, interquartile range, 95% confidence interval) were used to describe the baseline characteristics of the patients. Categorical variables were compared between groups using the χ^2^ test or Fisher’s exact test, as appropriate. Continuous variables were compared using the non-parametric Mann–Whitney *U*-test. Only values of *P* < 0.05 were considered significant. Variables associated with significant hepatic steatosis or liver fibrosis were investigated by logistic regression, while those associated with CAP values or liver stiffness were studied by linear regression analysis. To avoid overfitting and multicollinearity, variables were pre-selected based on clinical relevance and correlation analysis. Multicollinearity was formally excluded using variance inflation factor diagnostics (all VIF < 2.0). Correlation among variables was studied by Spearman test. IBM SPSS software was used for statistical analysis.

## Results

### Study cohort

Overall, a total of 274 PLWH underwent transient elastography during the study period. Nine patients were excluded from the analysis: eight owing to technical failure of the transient elastography exam with the M probe (6/8 with BMI > 30 kg/m^2^) and one (presenting S2 steatosis) due to missing clinical and laboratory data. Therefore, a total of 265 patients were included in our study (Table 1). Most patients were male (72%) with a median age of 53.9 years (interquartile range, IQR 46.9–60.7). The most frequent route of HIV transmission was through sexual intercourse (35.1% MSM, 34.7% heterosexual subjects) while 6.4% of patients had a history of intravenous drug use. Among comorbidities, hypertension was the most frequently reported (30.9%), followed by chronic kidney diseases (17.7%), peripheral vascular disease (11.3%) and diabetes (7.9%). Regarding coinfections, 33 patients (12.5%) were previously treated for hepatitis C with demonstrated sustained virological response (SVR). HBsAg was positive in 7 patients (2.6%), all with undetectable plasma HBV-DNA. Concurrent therapies included a statin in 31 patients (11.7%) and other lipid-lowering drugs in 28 patients (10.6%).

**Table 1.** Population’s characteristics (*n* = 265)

Variables	*n* (%) or median (IQR)
Age (years)	53.9 (46.9–60.7)
Female gender	74 (27.9)
Not Italian born	72 (27.2)
Risk factors for HIV infection	
Heterosexual	92(34.7)
MSM	93 (35.1)
IDU	17 (6.4)
Other/unknown	63 (23.8)
Comorbidities	
Diabetes	21 (7.9)
Hypertension	82 (30.9)
Ischaemic heart disease	7 (2.6)
Previous cerebrovascular disease	6 (2.3)
Peripheral vascular disease	30 (11.3)
Chronic kidney disease	47 (17.7)
Cancer	10 (3.8)
MASLD	113 (44.5)
Coinfections	
HBV (HbsAg+)	7 (2.6)
Eradicated HCV (HCV Ab+)	33 (12.5)
Concomitant medications	
Statin	31 (11.7)
Other lipid-lowering drugs	28 (10.6)
Antihypertensive drugs	71 (26.8)
Viro-immunological parameters	
Years of HIV infection	15.3 (8.7–24.3)
CD4 nadir (cells/µL)	176 (58–327)
Zenith viral load (log copies/mL)	5.1 (4.5–5.6)
AIDS-defining events	45 (17.0)
Currently on ART	261 (98.5)
Years since first ART	11.9 (7.9–17.8)
No. of therapeutic lines	5 (3–7)
HIV-RNA < 50 copies/mL	237 (89.4)
CD4, cells/mmc	758 (522–1052)
CD4/CD8 ratio	0.91 (0.6–1.3)
Type of ART	
InSTI-based	104 (39.2)
PI-based	14 (5.3)
NNRTI-based	44 (16.6)
Dual-based^a^	93 (35.1)
Other regimens	10 (3.8)
TAF use	138 (52.1)
Cumulative ART exposure (years)	
Thymidine analogues	0 (0–4.5)
NRTIs	10.8 (6.2–16.8)
TAF	4.0 (0.53–6.0)
NNRTIs	1.3 (0–7.8)
PIs	1.1 (0–7.4)
InSTIs	4.0 (0.6–7.2)
Chemistry	
Total cholesterol (mg/dL)	185 (155–208)
Triglycerides (mg/dL)	98 (73–138)
HDL (mg/dL)	50 (40–62)
LDL (mg/dL)	115 (93–137)

Ab, antibodies; AIDS, acquired immunodeficiency syndrome; ART, antiretroviral therapy; HbsAg, hepatitis B surface antigen; HBV, hepatitis B virus; HCV, hepatitis C virus; HDL, high-density lipoprotein; IDU, injecting drug users; InSTI, integrase strand transfer inhibitors; LDL, low-density lipoprotein; NNRTI, non-nucleoside reverse transcriptase inhibitors; NRTI, nucleoside reverse transcriptase inhibitors; PI, protease inhibitors; TAF, tenofovir alafenamide.

^a^Dual therapy was defined as a regimens including two drugs only: *n* = 65 dolutegravir/lamivudine, *n* = 17 InSTI + NNRTI, *n* = 10 PI + other drug and *n* = 1 other combination.

Concerning HIV variables, PLWH showed a long history of HIV infection (median time from diagnosis 15.3 years) and ART exposure (median 11.9 years), with AIDS-defining events reported in 45 patients (17%). At baseline, most patients (98.5%) were on ART (39.2% on InSTI-based, 16.6% on NNRTI-based, 5.3% on PI-based regimens, 35.1% on a dual therapy; tenofovir alafenamide was included in the backbone in 52.1%). Overall, 89.4% of PLWH had undetectable viral load (HIV-RNA < 50 copies/mL) with a median CD4 cell count of 758 cells/mmc and a median CD4/CD8 ratio of 0.91.

### Lifestyle and metabolic profile

Lifestyle and metabolic profile of our population is reported in Table 2. Analysis of the lifestyle questionnaire revealed that 42.6% of patients (*n* = 113) were active smokers, while 15.5% (*n* = 41) reported a regular alcohol intake (defined as daily alcohol consumption). Regarding physical activity, 40.4% (*n* = 107) maintained a regular exercise habit (defined as exercising twice a week or more), whereas 46.8% (*n* = 124) reported having a sedentary job.From a metabolic perspective, the median BMI was 25.3 kg/m^2^, with 12.8% of subjects showing obesity. Anthropometric abnormalities were frequently observed: a pathological waist circumference (>102 cm in men and >88 cm in women) was detected in 107 patients (40.4%) and a pathological waist-to-hip ratio (>0.90 in men and >0.85 in women) was detected in 226 patients (85.3%), demonstrating high prevalence of central fat accumulation.

**Table 2.** Lifestyle and metabolic profile of patients (*n* = 265)

Variables	*n* (%) or median (IQR)
Lifestyle variables	
Smokers	113 (42.6)
Daily alcohol intake	41 (15.5)
Regular physical activity	107 (40.4)
Sedentary work	124 (46.8)
Family history of cardiovascular disease	48 (18.1)
Metabolic parameters	
BMI (kg/m^2^)	25.3 (22.6–27.4)
BMI > 30 kg/m^2^	34 (12.8)
Waist circumference (cm)	92 (82–104)
Pathological waist circumference^a^	107 (40.4)
Waist/hip ratio	0.97 (0.9–1.0)
Pathological waist/hip ratio^b^	226 (85.3)
Metabolic syndrome	51 (19.2)
Mean blood pressure (mmHg)	100 (95–105)
High blood pressure^c^	97 (36.6)
Cardiovascular risk	
10-year CV risk categories—ASCVD (*n* = 253)	
Low (<5%)	125 (47.2)
Borderline (5%–7.4%)	39 (14.7)
Intermediate (7.5%–19.9%)	70 (26.4)
High (>20%)	19 (7.2)
Unknown	12 (4.5)
HOMA score (*n* = 248)	2.0 (1.19–4.23)
Insulin resistance^d^	125 (47.2)

ASCVD, atherosclerotic cardiovascular disease; CV, cardiovascular; HOMA, homeostatic model assessment.

^a^Males > 102 cm, females > 88 cm.

^b^Males > 0.90, females > 0.85.

^c^PA > 130/85 mmHg.

^d^Defined in presence of HOMA score value > 2.5.

The median 10-year cardiovascular risk (assessed by the ASCVD risk calculator) was 5.0% (IQR 2.3–11.0), with 33.6% of the cohort falling into the intermediate-to-high risk categories. Additionally, insulin resistance was identified in 47.2% of the population, with a median HOMA score of 2.0.

### Liver steatosis assessment

VCTE assessment yielded a median CAP value of 258 dB/m (IQR 216–300). Mild S1, moderate S2 and severe S3 steatosis were observed in 33 (12.5%), 53 (20%) and 76 (28.5%) PLWH, respectively, while no relevant steatosis (S0) was detected in 103 (38.9%) individuals.

Spearman’s correlation test identified significant positive associations between CAP and several parameters, including BMI (*r* = 0.573, *P* < 0.001), waist circumference (*r* = 0.462, *P* < 0.001), waist/hip circumference ratio (*r* = 0.356, *P* < 0.001), ASCVD score (*r* = 0.297, *P* < 0.001), HOMA score (*r* = 0.450, *P* < 0.001), total cholesterol (*r* = 0.120, *P* < 0.001) and triglycerides (*r* = 0.273, *P* < 0.001).

### Factors associated with severe steatosis (S3)

When compared to those with no/mild/moderate steatosis (S0–S2) (Table S1, available as Supplementary data at *JAC* Online), patients with severe S3 steatosis exhibited a significantly worse metabolic profile characterized by a significantly higher BMI (27.4 kg/m^2^ versus 24.2 kg/m^2^, *P* < 0.001), waist circumference (106 cm versus 87 cm, *P* < 0.001) and waist/hip circumference ratio (1.01 versus 0.95, *P* < 0.001); a higher prevalence of hypertension (40.4% versus 22.8%, *P* = 0.006) and of metabolic syndrome (29% versus 22%, *P* < 0.001); and a higher level of fasting glucose (96 versus 87 mg/dL, *P* < 0.001), LDL cholesterol (123 mg/dL versus 112 mg/dL, *P* = 0.012) and triglycerides (108 mg/dL versus 94 mg/dL, *P* = 0.006).

We analysed factors independently associated with severe steatosis by multivariate logistic regression (Table 3). After adjusting for several confounders, multiple independent variables associated with S3 steatosis were identified: longer cumulative exposure to integrase inhibitors (aOR 1.19 for +1 year, *P* = 0.002), a higher BMI (aOR 1.45 for +1 kg/m^2^, *P* < 0.001) and daily alcohol consumption (aOR 2.41, *P* = 0.031) and higher LDL values (aOR 1.20 for +10 mg/dL, *P* = 0.003). Interestingly, no association was observed with current InSTI treatment, whether combined with tenofovir alafenamide or not.

**Table 3.** Variables associated to severe S3 steatosis (univariable and multivariable logistic regression analysis)

Variables	Univariable analysis		Multivariable analysis	
	OR (95% CI)	*P*	aOR (95% CI)	*P*
Age, per +10 years	1.27 (0.99–1.64)	0.063		
Female gender	0.54 (0.28–1.01)	0.062		
Risk factors for HIV infection				
Heterosexual	1.09 (0.58–2.08)	0.779		
MSM	0.83 (0.25–2.77)	0.755		
IDU	1.25 (0.62–2.52)	0.538		
Other/unknown				
Comorbidities				
Diabetes	1.64 (0.6–4.9)	0.324		
Hypertension	2.80 (1.60–4.90)	**<0.001**	2.29 (0.94–5.57)	0.069
Previous ischaemic heart attack	1.90 (0.41–8.70)	0.408		
Previous stroke or TIA	1.25 (0.22–6.97)	0.799		
Peripheric vascular disease	1.51 (0.68–3.35)	0.307		
Chronic kidney disease	1.36 (0.70–2.66)	0.371		
Cancer	1.07 (0.27–4.25)	0.925		
Coinfections				
HBV (HBsAg+)	1.00 (0.19–5.24)	0.995		
Eradicated HCV (HCV Ab+)	1.09 (0.49–2.42)	0.826		
Viro-immunological status				
Years of HIV infection, per +10 years	1.57 (0.82–1.36)	0.668		
HIV-RNA < 50 copies/mL	1.01 ()0.42–2.39)	0.989		
CD4, per +100 cells/mmc	1.00 (0.94–1.07)	0.986		
Cumulative ART exposure:				
Thymidine analogues, per +1 year	0.98 (0.94–1.03)	0.494		
NRTI, per +1 year	1.01 (0.98–1.04)	0.450		
TAF, per +1 year	0.97 (0.88–1.08)	0.607		
NNRTI, per +1 year	0.966 (0.92–1.02)	0.185		
PI, per +1 year	1.01 (0.97–1.05)	0.572		
InSTI, per +1 year	1.09 (1.01–1.17)	**0.020**	1.19 (1.07–1.32)	**0**.**002**
Current ART				
InSTI + TAF-based	Ref	0.454		
InSTI without TAF	1.29 (0.67–2.48)	0.889		
NNRTI-based	0.84 (0.43–2.10)	0.165		
PI-based	0.34 (0.07–1.57)	0.987		
Other	1.01 (0.43–2.38)			
Metabolic findings				
BMI, per +1 kg/m^2^	1.42 (1.28–1.56)	**<0**.**001**	1.45 (1.27–1.65)	**<0**.**001**
Waist/hip circumference ratio	4292 (137–134 565)	**<0**.**001**	18.26 (0.18–18.58)	0.218
Lifestyle factors				
Daily alcohol consumption	2.05 (1.16–3.61)	**0**.**013**	2.41 (1.08–5.37)	**0**.**031**
Regular physical activity	0.50 (0.27–0.92)	**0**.**026**	0.58 (0.26–1.29)	0.180
Sedentary work	2.02 (1.18–3.47)	**0**.**011**	1.75 (0.83–3.71)	0.142
Laboratory findings				
Triglycerides per +10 mg/dL	1.00 (1.00–1.01)	**0**.**027**	1.02 (0.96–1.08)	0.581
HDL per +10 mg/dL	0.97 (0.96–0.99)	**0**.**005**	0.98 (0.72–1.31)	0.867
LDL per +10 mg/dL	1.11 (1.02–1.20)	**0**.**011**	1.20 (1.07–1.36)	**0**.**003**

ART, antiretroviral therapy; HDL, high-density lipoprotein; IDU, injecting drug users; InSTI, integrase strand transfer inhibitor; LDL, low-density lipoprotein; NNRTI, non-nucleoside reverse transcriptase inhibitors; NRTI, nucleoside reverse transcriptase inhibitors; PI, protease inhibitors; TAF, tenofovir alafenamide; TIA, transient ischaemic attack. Bold values indicate statistically significant *p*-values.

Linear regression analysis further confirmed that BMI (mean change = +6 db/m per 1 kg/m^2^, *P* < 0.001), waist-to-hip ratio (mean change = +87 db/m per unit, *P* = 0.021), daily alcohol consumption (mean change = +15 db/m, *P* = 0.019) and higher LDL levels (mean change = +3 db/m per +10 mg/dL, *P* = 0.002) were independently associated with increased CAP. Conversely, regular physical activity was significantly associated with a reduction in CAP (mean change = −20 db/m, *P* = 0.002) (Table S2).

### Liver stiffness measurements and liver fibrosis

Median liver stiffness was 4.70 kPa (IQR 4.0–5.9). Significant liver fibrosis (stiffness > 8 kPa) was observed in 25 (9.4%) PLWH.

We analysed factors associated with liver stiffness by linear regression analysis (Table 4). At a multivariate analysis, CAP values (mean change +0.07 kPa per +10 db/m, *P* = 0.016), higher BMI (mean change +0.10 kPa per +1 kg/m^2^, *P* = 0.021) and a longer time from HIV diagnosis (mean change +0.34 kPa for +10 years, *P* = 0.016) were independently associated with higher liver stiffness. In contrast, female gender (mean change −0.79 kPa, *P* = 0.031) and virological suppression (HIV-RNA < 50 copies/mL) were associated with a significantly reduced liver stiffness (mean change −1.61 kPa, *P* = 0.001).

**Table 4.** Variables associated with liver fibrosis measured through liver stiffness (kPa) (linear regression)

Variables	Univariable analysis		Multivariable analysis	
	Mean change (95% CI)	*P*-value	Adjusted mean change (95% CI)	*P*-value
Age, per +10 years	0.16 (−0.12/0.45)	0.250		
Female gender	−0.79 (−1.46/−0.12)	**0**.**021**	−0.79 (−1.50/−0.07)	**0**.**031**
Non Italian born	−0.09 (−0.77/0.60)	0.802		
IDU	0.87 (−0.34/2.08)	0.157		
Comorbidities				
Diabetes	1.24 (0.13/2.36)	**0.029**	0.73 (−0.35/1.81)	0.183
Hypertension	0.60 (−0.06/1.26)	0.072		
Previous IHA	2.79 (0.92/4.65)	**0.004**	1.42 (−0.34/3.18)	0.113
Previous stroke or TIA	−0.21 (2.25/1.84)	0.843		
Peripheral vascular disease	0.04 (−0.93/1.00)	0.941		
CKD	0.18 (−0.62/0.98)	0.658		
Cancer	−0.72 (−2.32/0.87)	0.374		
Coinfections				
HBV (HBsAg+)	0.46 (−1.44/2.35)	0.636		
Eradicated HCV (HCV Ab+)	0.86 (−0.05/1.78)	0.065		
Viro-immunological status				
Years of HIV infection, per +10 years	0.33 (0.04/0.62)	**0**.**025**	0.34 (0.06/0.62)	**0**.**016**
CD4, per +100 cell/mmc	−0.04 (−0.11/0.04)	0.357		
HIV-RNA < 50copies/mL	−1.27 (−2.25/−0.29)	**0**.**011**	−1.61 (−2.52/−0.70)	**0**.**001**
Cumulative ART exposure				
Thymidine analogues, per +1 year	0.00 (−0.05/0.06)	0.864		
NRTI, per +1 year	−0.01 (−0.04/0.03)	0.788		
TAF, per +1 year	0.04 (−0.09/0.16)	0.571		
NNRTI, per +1 year	0.04 (−0.02/0.09)	0.189		
PI, per +1 year	0.04 (−0.01/0.09)	0.116		
InSTI, per +1 year	−0.00 (−0.09/0.08)	0.925		
Metabolic parameters				
BMI, per +1 kg/m^2^	0.17 (0.10/0.25)	**<0**.**001**	0.10 (0.02/0.19)	**0**.**021**
Waist/hip circumference ratio	6.47 (3.29/9.65)	**<0**.**001**	0.68 (−3.04/4.40)	0.718
Lifestyle habits				
Daily alcohol intake	−0.01 (−0.86/0.84)	0.983		
Regular physical activity	−0.89 (−1.52/−0.25)	**0**.**006**	−0.47 (−1.07/0.13)	0.121
Sedentary work	0.42 (−0.19/1.03)	0.173		
CAP, per +10 db/m	0.13 (0.08/0.18)	**<0**.**001**	0.07 (0.01/0.13)	**0**.**016**

CAP, controlled attenuation parameter; CKD, chronic kidney disease; IDU, injecting drug users; IHA, ischaemic heart attack; InSTI, integrase strand transfer inhibitors; NNRTI, non-nucleoside reverse transcriptase inhibitors; NRTI, nucleoside reverse transcriptase inhibitors; PI, protease inhibitors; TAF, tenofovir alafenamide; TIA, transient ischaemic attack. Bold values indicate statistically significant *p*-values.

## Discussion

HIV infection is nowadays considered a chronic condition that can be managed in outpatient care, similarly to many other long-term diseases. In virologically suppressed and immunologically reconstituted patients on stable ART, quality of life is often comparable to that of individuals without HIV.^19^ However, longer survival among PLWH has led to new challenges, particularly the growing burden of metabolic and liver comorbidities. In recent years, hepatic steatosis and fibrosis have become increasingly common, raising concerns about long-term liver health in this population.

In our study, we performed a comprehensive assessment of the metabolic profile in a cohort of PLWH and we explored the prevalence and variables associated with hepatic steatosis and fibrosis, to identify strategies for monitoring and preventing liver disease progression in this population.

We observed a high prevalence of moderate/severe hepatic steatosis, affecting nearly half of the cohort (49% of patients with S2–S3 steatosis), consistent with findings from previous European studies.^13,20^ Importantly, patients with severe steatosis (S3) showed a markedly worse metabolic profile, documented by a significantly higher body weight, BMI and waist and hip circumferences and a greater prevalence of metabolic syndrome and hypertension. Moreover, lipid and glucose profiles were worse in patients with S3 steatosis. These results therefore show a high prevalence of metabolic risk factors within our cohort and reinforce existing literature demonstrating the significant prevalence of MASLD among PLWH.^21^ Careful monitoring of body weight and patient education regarding a healthy lifestyle are therefore essential, as recommended by all major guidelines.^12^

Among all evaluated variables, a longer history of InSTI exposure was significantly associated with S3 steatosis. Notably, each additional year of InSTI exposure was associated with a 19% increase in the odds of having S3 steatosis, independently of current BMI. This finding suggests that integrase inhibitors may contribute over time to hepatic changes leading to the development of hepatic steatosis. However, whether this association reflects a direct intrahepatic drug mechanism or an indirect effect mediated by prior cumulative InSTI-induced weight gain cannot be definitively established due to the cross-sectional study design.

In recent years, several studies have suggested an association between InSTI use, particularly dolutegravir and bictegravir, with significant weight gain and increases in visceral adipose mass.^3,5,13^ Analyses conducted in European and US cohorts have shown a higher prevalence or progression of hepatic steatosis in patients treated with InSTIs compared with non-InSTI regimens.^4,22^ Fernandes *et al.* recently reported a significant association between InSTI use and increased non-invasive indices of steatosis and fibrosis,^23^ consistent with our findings. Furthermore, according to published data, this effect appears amplified in the presence of tenofovir alafenamide, obesity or metabolic syndrome.^6,24^ Notably, cumulative tenofovir alafenamide exposure was not associated with severe steatosis in our cohort, in contrast to other reports.^25^

While the precise mechanisms linking InSTIs to hepatic steatosis are not yet fully elucidated, several pathways have been proposed, including increased caloric intake due to appetite stimulation, dysregulation of adipocyte differentiation, mitochondrial dysfunction, chronic low-grade systemic inflammation and the impairment of insulin signaling.^7^ Importantly, our findings suggest a cumulative effect of InSTI exposure rather than a simple association with the current regimen. This observation may have significant clinical implications, as prolonged use over many years could progressively increase the burden of liver disease in ageing PLWH. Although InSTIs remain essential agents in contemporary HIV treatment due to their high virological efficacy, durability and tolerability in both two- and three-drug regimens,^26^ our data support the need for regular and careful metabolic and hepatic monitoring in patients receiving long-term InSTI therapy, particularly among those with concomitant risk factors for MASLD such as obesity, dyslipidaemia or insulin resistance.

In addition to InSTI exposure, the association between severe steatosis and BMI confirms the relevant role of excess body weight, particularly adipose tissue accumulation, in visceral and hepatic fat deposition. Moreover, higher LDL cholesterol levels independently predicted severe steatosis, supporting the central role of lipid dysregulation in hepatic fat accumulation. This finding partially differs from that reported by Guaraldi *et al.*,^27^ who did not observe a significant association between LDL and steatotic liver disease, but identified lower HDL levels and a higher total cholesterol/HDL ratio as correlates of severe hepatic steatosis.

Furthermore, regular alcohol consumption remained associated with steatosis (even after excluding subjects with excessive alcohol consumption), suggesting that even low-to-moderate alcohol exposure may synergize with metabolic dysfunction in promoting liver fat deposition. On the opposite, regular physical activity was significantly associated with lower CAP values, confirming the protective role of exercise in reducing visceral adiposity and improving insulin sensitivity.

Overall, these results confirm that anthropometric and behavioural factors play a key role in modulating hepatic steatosis and underline the importance of lifestyle interventions, particularly weight control and regular physical activity, in the prevention and management of MASLD. This finding confirms previous literature showing that regular physical activity is an effective non-pharmacological therapeutic strategy to reduce the risk of disease progression.^28^ The latest EACS guidelines recommend at least 150 min of physical exercise per week among therapeutic interventions for MASLD management.^12^

A particularly relevant finding of our study was the strong association between steatosis and higher BMI with liver fibrosis. Patients with higher CAP values had a significantly higher risk of increased liver stiffness, independently of other metabolic and HIV-related variables. This observation is clinically important because it reinforces the concept that hepatic steatosis should not be considered a benign condition in PLWH. Instead, severe steatosis may represent an early stage in a progressive pathway leading to clinically significant liver damage. Recent longitudinal studies have demonstrated that both MASLD and weight gain significantly contribute to liver fibrosis progression in PLWH, independently of viral hepatitis coinfection.^29,30^

Longer duration of HIV infection was also independently associated with liver fibrosis, suggesting that chronic HIV-related inflammation and prolonged drug exposure may contribute to progressive liver damage over time. This finding is consistent with that reported by Mohr *et al*., who identified an association between HIV duration > 10 years and severe hepatic fibrosis in a cohort of PLWH stably treated with ART.^31^

Conversely, virological suppression was strongly associated with a lower liver stiffness. Given the chronic nature of fibrosis, single-point HIV-RNA suppression likely acts as a surrogate marker for sustained, long-term viral control in this long-treated cohort (median ART duration 11.9 years), rather than an acute determinant. Overall, these findings support the beneficial effect of effective ART in reducing liver-related complications^32^ and further underline the importance of early HIV diagnosis, prompt ART initiation and durable virological suppression to improve both HIV outcome and liver health. Nevertheless, interpreting non-invasive fibrosis markers in an unselected HIV population warrants caution. In low-prevalence settings, unselective screening carries a risk of false positives, which can be further exacerbated by high CAP values artificially elevating liver stiffness. For this reason, current guidelines advocate for a sequential, risk-stratified approach (using simple tools like FIB-4 as a first-line filter) rather than universal elastographic screening.^12^

We recognize that our study can have some limitations that should be considered when interpreting the results. The cross-sectional design does not allow definitive conclusions regarding causality between InSTI exposure and hepatic steatosis or fibrosis progression. Specifically, while our multivariable analysis adjusted for current BMI, the lack of longitudinal historical weight data prior to VCTE evaluation prevented us from performing a formal mediation analysis. Consequently, we cannot rule out that the observed association between cumulative InSTI exposure and severe steatosis is partially or largely mediated by InSTI-induced weight gain over time, rather than representing an entirely direct drug effect. Second, liver biopsy was not performed, and hepatic assessment relied on non-invasive elastography-based methods. However, VCTE and CAP are validated and widely recommended tools for the non-invasive assessment of steatosis and fibrosis in PLWH. Universally agreed CAP thresholds for grading hepatic steatosis in PLWH are still lacking. For instance, the landmark ECHAM study established a cut-off of 280 dB/m to define moderate-to-severe steatosis (S ≥ 2) in HIV-monoinfected individuals,^33^ whereas higher thresholds are typically employed to capture severe steatosis (S3). Nevertheless, the validity of the cut-off adopted in our cohort is supported by its consistent correlation with classical metabolic risk factors.

Thirdly, a technical limitation of our study is that transient elastography was performed exclusively using the M probe, as the XL probe was unavailable. While the lack of an XL probe can lead to technical failure in patients with obesity, thus potentially underestimating MASLD prevalence, this limitation appears to have had a minimal impact on our cohort. Indeed, only eight patients (2.9% of the overall population) were excluded due to unsuccessful liver stiffness and CAP measurements, suggesting that selection bias was substantially contained. Additionally, physical activity was self-reported using a questionnaire that evaluated frequency rather than standardized intensity (e.g. moderate-to-vigorous physical activity) or duration. While this qualitative assessment represents a study limitation, incorporating physical activity frequency, a lifestyle variable often overlooked in similar studies, still provides valuable insights into our cohort. Furthermore, detailed nutritional information was not collected in our cohort. Although the lack of dietary data prevents us from accounting for specific caloric or nutrient intake, a well-established lifestyle factor in MASLD, this is a frequent limitation in observational real-world studies, where comprehensive dietary tracking is often unfeasible. Additionally, the cross-sectional design limited viral load assessment to a single time point. While this reflects current viral control—serving as a proxy for long-term ART efficacy—systematic longitudinal viremia data were not fully available to adequately quantify continuous cumulative suppression. Finally, this was a single-centre study, and external validation in larger multicenter cohorts will be important.

In conclusion, hepatic steatosis and liver fibrosis were highly prevalent in this cohort of PLWH receiving ART. Metabolic dysfunction, obesity and cumulative InSTI exposure emerged as major determinants of severe steatosis, while severe steatosis itself was strongly associated with clinically fibrosis. These findings support the hypothesis that prolonged metabolic alterations, chronic ART exposure and chronic HIV-related inflammation may contribute to progressive liver disease in PLWH. Early identification of steatosis and fibrosis, together with targeted lifestyle and metabolic interventions, should therefore become an integral component of long-term HIV care.

## Supplementary Material

dkag351_Supplementary_Data

## Supplementary data

Tables S1 and S2 are available as Supplementary data at *JAC* Online.
