# Supplementary material for: Cumulative exposure to integrase inhibitors and metabolic factors as independent predictors of severe hepatic steatosis in people with HIV
Source: J Antimicrob Chemother. 2026 Oct 21;81(11):dkag351. doi: 10.1093/jac/dkag351 (PMC13684268; doi:10.1093/jac/dkag351)
Supplement: Supplementary file 1 [file dkag351_supplementary_data.docx]

**Table S1:** Comparison of patients with S0-2 vs S3 steatosis

| **Variables** | **S0-S2**  **(n = 189)** | **S3**  **(n = 76)** | **p-value**  **(<0.05)** |
| --- | --- | --- | --- |
| **Age (years)** | 53.9 (45.5-60.0) | 55 (49-63) | 0.084 |
| **Female gender** | 59 (31.2) | 15 (19.7) | 0.060 |
| **Not Italian born** | 52 (27.5) | 20 (26.3) | 0.843 |
|  | | | |
| **Risk factors for HIV infection:** |  |  | 0.892 |
| Heterosexual  MSM  IDU  Other/unknown | 67 (35.4)  66 (34.9)  13 (6.9)  43 (22.8) | 25 (32.9)  27 (35.5)  4 (5.3)  20 (26.3) |  |
| **Comorbidities:** | | | |
| Diabetes  Hypertension  Previous ischemic heart disease  Previous stroke or TIA  Peripheric vascular disease  Chronic kidney disease  Cancer | 13 (6.9)  46 (24.3)  4 (2.1)  4 (2.1)  19 (10.1)  31 (16.4)  7 (3.7) | 8 (10.5)  36 (47.4)  3 (3.9)  2 (2.6)  11 (14.5)  16 (21.1)  3 (3.9) | 0.320  **<0.001**  0.413  1.000  0.304  0.370  1.000 |
| **Coinfections:** |  |  |  |
| HBV (HbsAg+) | 5 (2.6) | 2 (2.6) | 0.999 |
| Eradicated HCV (HCV Ab+) | 23 (12.2) | 10 (13.2) | 0.272 |
| **Concomitant medications:** | | | |
| Statin  Other oral lowering lipid drugs | 20 (10.6)  16 (8.59) | 11 (14.5)  12 (15.8) | 0.373  0.079 |
|  |  |  |  |
| **Viro-immunological parameters:** | | | |
| Years from HIV infection | 15.1 (8.6-24.7) | 15.6 (9.4-23.6) | 0.620 |
| Nadir CD4 (cells/µl) | 190 (81-352) | 136 (42-287) | 0.058 |
| Zenith HIV-RNA (log copies/ml) | 5.0 (4.5-5.6) | 5.26 (4.67-5.81) | 0.231 |
| AIDS-defining events | 30 (15.9) | 15 (19.7) | 0.449 |
| Years from first ART | 12.3 (7.8-17.8) | 11.4 (8.3-2.0) | 0.951 |
| On ART | 186 (98.4) | 75 (98.7) | 1.000 |
| HIV-RNA < 50 copies/ml | 169 (89.4) | 68 (89.5) | 0.989 |
| CD4 cell count | 7.5 (5.5-10.3) | 763 (488-1093) | 0.773 |
| CD4/CD8 ratio | 0.90 (0.63-1.23) | 0.94 (0.47-1.36) | 0.630 |
|  | | |  |
| **Type of ART:** |  |  | 0.791 |
| InSTI-based  PI-based  NNRTI-based  Dual-based  Other regimens | 72 (38.1)  12 (6.3)  32 (16.9)  66 (34.9)  7 (3.7) | 32 (42.1)  14 (5.3)  44 (16.6)  93 (35.1)  10 (3.8) |  |
| TAF use | 101 (53.4) | 37 (48.7) | 0.483 |
| **Metabolic parameters:** | | | |
| BMI (kg/m^2^) | 24.2 (21.6-26.2) | 27-4 (25.9-30.1) | **<0.001** |
| BMI >30 (obesity) | 9 (4.8) | 25 (32.9) | **<0.001** |
| Waist circumference (cm) | 87 (80-96) | 106 (98-115) | **<0.001** |
| Pathological waist circumference | 55 (29.1) | 52 (68.4) | **<0.001** |
| Waist/hip circumference ratio | 0.95 (0.90-1.02) | 1.01 (0.96-1.06) | **<0.001** |
| Pathological waist/hip circumference ratio | 153 (81.0) | 73 (96.1) | **0.002** |
| Metabolic syndrome | 22 (11.6) | 29 (38.2) | **<0.001** |
|  |  |  |  |
| **ASCVD score:** |  |  | 0.134 |
| Low CV risk  Borderline CV risk  Intermediate CV risk  High CV risk  Unknown | 96 (50.8)  29 (15.39  45 (23.8)  10 (5.3)  9 (4.8) | 29 (38.2)  10 (13.2)  25 (32.9)  9 (11.8)  3 (3.9) |  |
|  |  |  |  |
| **Stiffness (kPa):** | 4.5 (3.9-5.3) | 5.4 (4.6-7.3) | **<0.001** |
| F0-F1  F2  F3  F4 | 170 (89.9)  7 (3.7)  4 (2.1)  8 (4.2) | 52 (68.4)  11 (14.5)  5 (6.6)  8 (10.5) | **<0.001** |
| **MASLD:** | 46 (24.3) | 72 (94.7) | **<0.001** |
| **Lifestyle questionnaire:** | | | |
| Smokers | 85 (45.0) | 28 (36.8) | **0.047** |
| Daily alcohol consumption | 28 (15.19 | 13 (18.3) | 0.524 |
| Regular physical activity | 84 (44.4) | 23 (30.3) | **0.033** |
| Sedentary work | 79 (41.8) | 45 (59.2) | **0.010** |
| Family history of CV diseases | 33 (17.5) | 15 (19.7) | 0.663 |
| **Chemistry:** | | | |
| GPT (UI/L) | 19 (15-25) | 27 (18-41) | **<0.001** |
| GGT (UI/L) | 18 (14-26) | 25 (19-49) | **<0.001** |
| Glucose (g/dL) | 87 (81-96) | 96 (86-104) | **<0.001** |
| HOMA score | 1.81 (0.92-3.01) | 3.57 (1.92-6.17) | **0.007** |
| Insulin resistance | 72 (38.1) | 53 (69.7) | **<0.001** |
| Total Cholesterol (mg/dl) | 182 (153-207) | 191 (161-208) | 0.192 |
| Triglycerides (mg/dl) | 94 (68-127) | 108 (84-171) | **0.006** |
| HDL (mg/dl) | 52 (41-64) | 46 (39-56) | **0.005** |
| LDL (mg/dl) | 112 (91-136) | 123 (102-145) | **0.012** |

**Abbreviations:** AIDS = Acquired Immunodeficiency Syndrome; ART = Antiretroviral Therapy; ASCVD = Atherosclerotic Cardiovascular Disease; BMI = Body Mass Index; CV = Cardiovascular; GGT = Gamma-Glutamyl Transferase; GOT = Glutamate Oxaloacetate Transaminase; GPT = Glutamate Pyruvate Transaminase; HDL = High-Density Lipoprotein; HOMA = Homeostatic Model Assessment; IDU = Injection Drug Users; INSTI = Integrase Strand Transfer Inhibitor; LDL = Low-Density Lipoprotein; MASLD = Metabolic Dysfunction-Associated Steatotic Liver Disease; MSM = Men Who Have Sex with Men; NNRTIs = Non-Nucleoside Reverse Transcriptase Inhibitors; NRTIs = Nucleoside Reverse Transcriptase Inhibitors; TAF = Tenofovir Alafenamide; TIA = Transient Ischemic Attack.

**Table S2:** Analysis of variables associated with CAP (linear regression).

| **Variables** | **Univariable analysis** | | **Multivariable analysis** | |
| --- | --- | --- | --- | --- |
|  | **Mean change (CI 95%)** | **p** | **Adjusted mean change (CI 95%)** | **p** |
| Age (years) per +10 years | 13 (7 / 20) | **<0.001** | 4 (-5 / 12) | 0.388 |
| Female gender | -12 (-29 / 4) | 0.156 |  |  |
| IDU | -13 (-43 / 16) | 0.377 |  |  |
|  |  |  |  |  |
| **Comorbidities:** | | | | |
| Diabetes  Hypertension  Previous IHA  Previous stroke or TIA  Peripheral vascular disease  CKD  Cancer  **Coinfections:**  HBV (HbsAg+)  Eradicated HCV (HCV Ab+) | 31 (4 / 59)  43 (27 / 58)  55 (9 / 101)  0 (-46/55)  22 (-1 / 45)  0 (-8/31)  0 (-63/15)  0 (-52/41)  0 (-21/25) | **0.025**  **<0.001**  **0.019**  0.857  0.065  0.249  0.228  0.815  0.842 | -7 (-33 / 20)  17 (1 / 33)  16 (-21 / 53) | 0.625  **0.035**  0.391 |
| **Viro-immunological status**: | | | | |
| Years since HIV infection, per +1 year | 4 (-2/12) | 0.196 |  |  |
| HIV-RNA <50 copies/mL | 9 (-15 / 33) | 0.467 |  |  |
| CD4, per +100 cells/mmc | 1 (-1/3) | 0.181 |  |  |
| Cumulative ART exposure:  -Thymidine analogues, per +1 year  -NRTI, per +1 year  -TAF, per +1 year  -NNRTI, per +1 year  -PI, per +1 year  -InSTI, per +1 year | 0 (-1/1)  0 (0/1)  1 (-2 / 4)  0 (-1/1)  1 (0/29  2 (-0 / 4) | 0.965  0.129  0.380  0.990  0.144  0.084 |  |  |
| **Metabolic findings:** | | | | |
| BMI, per +1 kg/m^2^ | 8 (7 / 10) | **<0.001** | 6 (5 / 8) | **<0.001** |
| Waist/hip circumference ratio | 249 (175 / 324) | **<0.001** | 87 (13 / 161) | **0.021** |
| **Lifestyle habits:** | | | | |
| Daily alcohol consumption | 18 (4 / 33) | **0.015** | 15 (2 / 27) | **0.019** |
| Regular physical activity | -30 (-45 /-14) | **<0.001** | -20 (-33 / -7) | **0.002** |
| Sedentary work | 21 (6 / 36) | **0.006** | 7 (-5 / 20) | 0.263 |
| **Laboratory findings:** | | | | |
| Triglycerides, per +10 mg/dL | 1 (1/2) | **<0.001** | 1 (-0/1) | 0.087 |
| HDL, per +10 mg/dL | -6 (-11/-1) | 0.011 | 3 (-2/7) | 0.279 |
| LDL, per + 10 mg/dL | 3 (0 / 5) | **0.021** | 3 (1 / 5) | **0.002** |

**Abbreviations:** ART = Antiretroviral Therapy; BMI = Body Mass Index; CKD= Chronic Kidney Disease, IHA=Ischemic Heart attack; InSTI = Integrase Strand Transfer Inhibitors; LDL = Low-Density Lipoprotein; NNRTIs = Non-Nucleoside Reverse Transcriptase Inhibitors; NRTIs = Nucleoside Reverse Transcriptase Inhibitors; PIs = protein inhibitors; TIA = Transient Ischemic Attack; TAF= tenofovir alafenamide.
